# The relationship of the immune response mediator genes’ polymorphic variants with the methotrexate efficacy in juvenile idiopathic arthritis

**DOI:** 10.3906/sag-1910-96

**Published:** 2020-06-23

**Authors:** Liliia Sh. NAZAROVA, Ksenia V. DANILKO, Viktor A. MALIEVSKY, Denis O. KARIMOV, Akhat B. BAKIROV, Tatiana V. VIKTOROVA

**Affiliations:** 1 Department of Toxicology and Genetics, Ufa Research Institute of Occupational Health and Human Ecology, Ufa Russia; 2 Central Research Laboratory, Bashkir State Medical University, Ufa Russia; 3 Department of Biology, Bashkir State Medical University, Ufa Russia; 4 Department of Hospital Pediatrics, Bashkir State Medical University, Ufa Russia; 5 Ufa Research Institute of Occupational Health and Human Ecology, Ufa Russia; 6 Department of Therapy and Occupational Diseases with the course of Institute of Additional Professional Education,Bashkir State Medical University, Ufa Russia

**Keywords:** Juvenile idiopathic arthritis, polymorphic loci, methotrexate efficacy, predictors

## Abstract

**Background/aim:**

The aim of the study was to analyze the relationship of the immune response mediator genes’ polymorphic loci (*TNFA* rs1800629, *LTA* rs909253, *IL1B *rs16944, *IL2-IL21* rs6822844, *IL2RA* rs2104286, *IL6* rs1800795, *IL10* rs1800872, *MIF* rs755622, *CTLA4* rs3087243, *NFKB1* rs28362491, *PTPN22* rs2476601, *PADI4* rs2240336) variants with the methotrexate efficacy in juvenile idiopathic arthritis (JIA).

**Materials and methods:**

The study included 274 JIA patients from the Republic of Bashkortostan, Russia. Achieving the American College of Rheumatology Pediatric 30 (ACR Pedi 30) response was regarded as the presence of the response to methotrexate (otherwise, as the absence), while achieving clinical remission on medication (Wallace et al., 2011) - as the sufficient response (otherwise, as the insufficient). Genotyping was conducted by the real-time polymerase chain reaction.

**Results:**

Associations with an altered risk of the nonresponse to methotrexate in JIA were observed for the alleles/genotypes of the loci* IL10* rs1800872 (in girls) and *NFKB1* rs28362491 (in girls); with an altered risk of the insufficient response to methotrexate in JIA – for the alleles/genotypes of the loci *IL1B *rs16944 (in boys), *CTLA4* rs3087243 (in boys), *NFKB1* rs28362491 (in girls) and the haplotype *TNFA* rs1800629*A - *LTA* rs909253*G (in girls).

**Conclusion:**

As a result of the study, the relationship of the alleles/genotypes of the *IL1B *rs16944, *IL10* rs1800872, *CTLA4* rs3087243, *NFKB1* rs28362491 polymorphic loci and the *TNFA* rs1800629*A - *LTA* rs909253*G haplotype with the methotrexate efficacy in JIA was established (taking into account the differences by sex).

## 1. Introduction

Juvenile idiopathic arthritis (JIA) is the most common childhood rheumatic disease, which begins before the age of 16 and is characterized by arthritis persisting at least 6 weeks [1–3]. The JIA diagnosis is entirely based on the clinical features, and the generally accepted disease classification is the International League of Associations for Rheumatology (ILAR) classification [1]. 

Being a chronic, steadily progressive disease, JIA can lead to short-term and long-term disability [2]. Thus, early and effective therapy is crucial for preventing JIA complications and patients’ disability [3–7].

Methotrexate is the most widely used disease-modifying antirheumatic drug in the JIA treatment [4]. Methotrexate was first described as the antimetabolite of folic acid and was used in the field of oncology, but the mechanism of its action in rheumatology is still poorly understood [8]. The antiinflammatory effects of methotrexate in JIA may be mediated by addition to folate antagonism mechanisms, such as adenosine release, inhibition of spermine/spermidine production and/or alteration of cellular redox state [9]. It has been shown that methotrexate may directly or indirectly affect the expression and/or secretion of the various immune response mediators involved in the pathogenesis of JIA and other autoimmune diseases, including cytokines (such as tumour necrosis factor alpha (TNFα), interleukin 1 (IL1), IL2, IL6, IL10), nuclear factor kappa B (NFκB), cytotoxic T-lymphocyte associated protein 4 (CTLA4) and others [10–13].

Nevertheless, the response to therapy varies among the JIA patients, and the identifying of early predictors of the methotrexate efficacy is highly relevant [4,7]. Currently, a search for the genetic predictors of the methotrexate efficacy in JIA is underway. The most promising are polymorphic variants of genes involved in the methotrexate metabolic pathway or genes, whose expression changes under the methotrexate effect, as well as a number of markers identified in the genome-wide association study (GWAS) [4,9,14–16]. In addition, the relationship can be assumed between the methotrexate efficacy and the polymorphic variants of other genes encoding proteins, presumably playing an important role in the JIA pathogenesis, including interleukin 2 receptor subunit alpha (IL2Rα), lymphotoxin alpha (LTα), macrophage migration inhibitory factor (MIF), protein tyrosine phosphatase nonreceptor type 22 (PTPN22), peptidyl arginine deiminase 4 (PADI4) [17–19].

Considering what is mentioned above, the search for the predictors of the JIA patients’ response to methotrexate among some polymorphic variants of genes, whose products are involved in the pathogenesis of the autoimmune diseases and are the potential targets for the methotrexate exposure, seems appropriate. Identification of these markers may provide an opportunity to timely predict the JIA therapy efficacy, to determine an adequate treatment strategy, and to prevent the development of the disease complications [3–7]. Thus, the aim of the study was to analyze the relationship of the immune response mediator genes polymorphic loci (*TNFA* rs1800629, *LTA* rs909253, *IL1B *rs16944, *IL2-IL21* rs6822844, *IL2RA* rs2104286, *IL6* rs1800795, *IL10* rs1800872, *MIF* rs755622, *CTLA4* rs3087243, *NFKB1* rs28362491, *PTPN22* rs2476601, *PADI4* rs2240336) variants with the methotrexate efficacy in JIA.

## 2. Materials and methods

### 2.1. Study design and subjects

A case-control study was conducted. The study was approved by the expert council on biomedical ethics of Bashkir State Medical University (Ufa, Russia).

The study included 274 JIA patients treated with methotrexate. Parents of all patients signed the voluntary informed consent. All patients were residents of the Republic of Bashkortostan and were examined and treated in the cardio-rheumatological department of the Republican Children’s Clinical Hospital (Ufa, Russia) in 2011–2017. The median age of the patients was 8.69 (4.82; 12.80) years; the ratio of boys/girls was 31.75%/68.25%. The JIA diagnosis was established according to the International League of Associations for Rheumatology (ILAR) criteria [1]. According to the ILAR criteria, the following JIA subtypes were presented: systemic arthritis (n = 25 (9.12%)), rheumatoid factor positive polyarthritis (n = 5 (1.82%)), rheumatoid factor negative polyarthritis (n  =  76 (27.74%)), persistent oligoarthritis (n  =  77 (28.10%)), extended oligoarthritis (n  = 41 (14.96%)), enthesitis related arthritis (n  = 26 (9.49%)), psoriatic arthritis (n  =  8 (2.92%)), undifferentiated arthritis (n = 16 (5.84%)).

Achieving the American College of Rheumatology Pediatric 30 (ACR Pedi 30) response was regarded as the presence of the response to methotrexate (otherwise, as the absence), while achieving clinical remission on medication (Wallace et al., 2011) - as the sufficient response (otherwise, as the insufficient) [5,20–22]. Evaluation of the presence of the response to methotrexate was performed in patients who received the therapy for at least 3 months, and the sufficiency of the response was evaluated in patients who received the therapy for at least 12 months.

### 2.2. Genotyping

DNA isolation from the lymphocytes of the whole blood samples was performed using a standard phenol-chloroform method [23]. Genotyping of all the patients for the 12 polymorphic loci (*TNFA* rs1800629, *LTA* rs909253, *IL1B *rs16944, *IL2-IL21* rs6822844, *IL2RA* rs2104286, *IL6* rs1800795, *IL10* rs1800872, *MIF* rs755622, *CTLA4* rs3087243, *NFKB1* rs28362491, *PTPN22* rs2476601, *PADI4* rs2240336) was conducted by the real-time polymerase chain reaction (StepOnePlus™ Real-Time PCR System, Applied Biosystems, USA). Sequence-specific primers and allele-specific probes were designed and synthesized by the “DNK-syntez” company (Russia). 

### 2.3. Statistical analysis

Statistical processing of the results was carried out using Microsoft Excel, SNPStats, R v.3.4.2 (R Core Team, 2017), PowerMarker v.3.25, STATISTICA v.10 (StatSoft, Inc.) [24–26]. 

To compare the genotype and allele frequencies in the studied groups the two-tailed Fisher’s exact test was used. A similar analysis was also performed separately for boys and girls. The multiple testing correction of the P-values was carried out by applying the permutation test with 104 permutes (pcor) [24,27]. The odds ratio (OR) with the Baptista–Pike exact conditional 95% confidence interval (95% CI) were also calculated [28].

The haplotypes of the *TNFA* rs1800629 and *LTA* rs909253 polymorphic loci, located in the same cluster on chromosome 6, have also been studied as the potential predictors of the methotrexate efficacy in JIA, since the linkage disequilibrium for these loci was almost complete (D’ = 0.9989, P = 0.000). The association between the *TNFA* rs1800629 - *LTA* rs909253 loci haplotypes and the methotrexate efficacy in JIA was analyzed using the logistic regression method [25].

The analysis of the inheritance models (codominant, each genotype can give a different and nonadditive risk; dominant, one copy of the variant allele is enough to change the risk; recessive, two copies of the variant allele are necessary to change the risk; overdominant, heterozygous genotype is compared to a pool of both homozygous genotypes; log-additive, each copy of the variant allele changes the risk in an additive form) was carried out using the logistic regression method, and the selection of the best one - using the Akaike’s Information Criterion. The inheritance model with the lowest Akaike’s Information Criterion value was considered as the model that best fits the data [25].

In all the cases the results were considered statistically significant at P < 0.05.

## 3. Results

### 3.1. The study of the predisposition to the absence of the response to methotrexate in JIA

When studying the *IL10* rs1800872 polymorphic locus, no predictors of the absence of the response to methotrexate were found in the whole JIA group, as well as in boys with JIA (P > 0.1) (Table 1). At the same time, in girls with JIA, the frequency of the* IL10* rs1800872*A allele was significantly higher, and of the *IL10* rs1800872*CС genotype – significantly lower in the absence of the response to methotrexate than in its presence (*A: 38.16% vs. 24.81%, P = 0.029, pcor = 0.027, OR = 1.87, 95% CI 1.069–3.162; *CC: 36.84% vs. 57.36%, P = 0.028, pcor = 0.029, OR = 0.434, 95% CI 0.215–0.932, respectively). The dominant model of inheritance reflected the results better than the others (CA+AA vs. CC, P = 0.026).

**Table 1:** The relationship of the polymorphic loci alleles and genotypes with the absence of the response to methotrexate in JIA patients.

Polymorphic loci			The whole group (f+m)				Female (f)				Male (m)				ni
Genes	rs	Alleles	Аlleles and genotypes frequencies, %		Model of inheritance		Аlleles and genotypes frequencies, %		Model of inheritance		Аlleles and genotypes frequencies, %		Model of inheritance		The total number of genotyped subjects, ni
			absence of responsepresence of response		var p	OR 95% CI	absence of responsepresence of response		var p	OR 95% CI	absence of responsepresence of response		var p	OR 95% CI	absence of response presence of response
		(1)/(2)	(2)	(11)/(12)/(22)			(2)	(11)/(12)/(22)			(2)	(11)/(12)/(22)			f+m : f : m
TNFA	1800629	G/A	7.8	84.5/15.5/0.0	NS	NS	7.9	84.2/15.8/0.0	-	-	7.5	85.0/15.0/0.0	NS	NS	58 : 38 : 20
			11.6	77.3/22.1/0.6			11.2	77.5/22.5/0.0			12.5	76.9/21.2/1.9			181 : 129 : 52
LTA	909253	A/G	36.2	36.2/55.2/8.6	NS	NS	35.5	39.5/50.0/10.5	NS	NS	37.5	30.0/65.0/5.0	NS	NS	58 : 38 : 20
			32.3	46.4/42.5/11.0			31.4	47.3/42.6/10.1			34.6	44.2/42.3/13.5			181 : 129 : 52
IL1B	16944	C/T	40.5	36.2/46.6/17.2	NS	NS	39.5	34.2/52.6/13.2	NS	NS	42.5	40.0/35.0/25.0	NS	NS	58 : 38 : 20
			36.5	41.4/44.2/14.4			37.6	38.8/47.3/14.0			33.7	48.1/36.5/15.4			181 : 129 : 52
IL2-21	6822844	G/T	11.2	81.0/15.5/3.4	rec§0.017	NA0.00-NA	10.5	81.6/15.8/2.6	NS	NS	12.5	80.0/15.0/5.0	NS	NS	58 : 38 : 20
			8.0	84.0/16.0/0.0			8.9	82.2/17.8/0.0			5.8	88.5/11.5/0.0			181 : 129 : 52
IL2RA	2104286	A/G	14.7	74.1/22.4/3.4	NS	NS	14.5	73.7/23.7/2.6	NS	NS	15.0	75.0/20.0/5.0	NS	NS	58 : 38 : 20
			16.6	69.6/27.6/2.8			17.1	69.0/27.9/3.1			15.4	71.2/26.9/1.9			181 : 129 : 52
IL6	1800795	G/C	37.1	36.2/53.4/10.3	NS	NS	35.5	34.2/60.5/5.3	NS	NS	40.0	40.0/40.0/20.0	NS	NS	58 : 38 : 20
			37.8	38.7/47.0/14.4			38.0	36.4/51.2/12.4			37.5	44.2/36.5/19.2			181 : 129 : 52
IL10	1800872	C/A	36.2	43.1/41.4/15.5	NS	NS	38.2	36.8/50.0/13.2	dom0.026	2.311.09-4.86	32.5	55.0/25.0/20.0	NS	NS	58 : 38 : 20
			28.7	52.5/37.6/9.9			24.8	57.4/35.7/7.0			38.5	40.4/42.3/17.3			181 : 129 : 52
MIF	755622	G/C	17.2	67.2/31.0/1.7	NS	NS	15.8	71.1/26.3/2.6	NS	NS	20.0	60.0/40.0/0.0	NS	NS	58 : 38 : 20
			19.9	64.1/32.0/3.9			20.2	62.8/34.1/3.1			19.2	67.3/26.9/5.8			181 : 129 : 52
CTLA4	3087243	G/A	29.3	50.0/41.4/8.6	NS	NS	25.0	57.9/34.2/7.9	NS	NS	37.5	35.0/55.0/10.0	NS	NS	58 : 38 : 20
			32.6	47.0/40.9/12.2			34.9	42.6/45.0/12.4			26.9	57.7/30.8/11.5			181 : 129 : 52
NFKB1	28362491	I/D	51.7	27.6/41.4/31.0	rec0.0032	2.941.46-5.94	51.3	28.9/39.5/31.6	rec0.018	2.851.22-6.63	52.5	25.0/45.0/30.0	NS	NS	58 : 38 : 20
			40.1	33.1/53.6/13.3			41.1	31.8/54.3/14.0			37.5	36.5/51.9/11.5			181 : 129 : 52
PTPN22	2476601	G/A	14.7	74.1/22.4/3.4	NS	NS	14.5	76.3/18.4/5.3	NS	NS	15.0	70.0/30.0/0.0	NS	NS	58 : 38 : 20
			13.0	75.7/22.7/1.7			14.0	73.6/24.8/1.6			10.6	80.8/17.3/1.9			181 : 129 : 52
PADI4	2240336	G/A	48.3	20.7/62.1/17.2	dom§0.032	2.101.04-4.24	47.4	23.7/57.9/18.4	NS	NS	50.0	15.0/70.0/15.0	NS	NS	58 : 38 : 20
			41.2	35.4/47.0/17.7			41.9	34.9/46.5/18.6			39.4	36.5/48.1/15.4			181 : 129 : 52

Note: (1) – the major allele, (2) – the minor allele; (11) and (22) – genotypes homozygous for the major and minor alleles, respectively; (12) – heterozygous genotype; f+m – female and male, f – female, m – male; Model of inheritance – the best model of inheritance (regression analysis): var – type of the model: dom – dominant: (12)+(22) vs (11); rec – recessive: (22) vs (12)+(11); statistically significant results are highlighted in bold; the § symbol indicates models, the differences in which did not reach the statistical significance level in the analysis using the two-tailed Fisher’s exact test; NS – statistically nonsignificant; NA – not available.

Analysis of the *NFKB1* rs28362491 polymorphic locus showed that the *NFKB1* rs28362491*DD genotype was significantly more common, and the *NFKB1* rs28362491*I allele was significantly less common in JIA patients with the absence of the response to methotrexate than in those with its presence (*DD: 31.03% vs. 13.26%, P = 0.005, pcor = 0.004, OR = 2.944, 95% CI 1.484–5.938; *I: 48.28% vs. 59.94%, P = 0.031, pcor = 0.033, OR = 0.624, 95% CI 0.410–0.944, respectively). After stratification based on the sex of the patients, similar significant differences for the *NFKB1* rs28362491*DD genotype were found only in girls with JIA (P = 0.017, pcor = 0.017, OR = 2.846, 95% CI 1.253–6.483), and only a similar trend was observed in boys with JIA (pcor = 0.085), which is probably due to the smaller sample size. The best inheritance model turned out to be the recessive (DD vs. II+ID, P = 0.0032 and P = 0.018 in JIA as a whole and in girls, respectively).

For the alleles and genotypes of the *TNFA* rs1800629, *LTA* rs909253, *IL1B* rs16944, *IL2-IL21* rs6822844, *IL2RA* rs2104286, *IL6* rs1800795, *MIF* rs755622, *CTLA4* rs3087243, *PTPN22* rs2476601, *PADI4* rs2240336 polymorphic loci, as well as haplotypes of the *TNFA* rs1800629 - *LTA* rs909253 loci, no associations were observed with the considered trait (P > 0.05). 

When studying the *PADI4* rs2240336 polymorphic locus, a borderline significance level tendency was observed for the increase of the proportion of the *PADI4* rs2240336*GA genotype and the decrease of the proportion of the *PADI4* rs2240336*GG genotype in JIA patients with the absence of the response to methotrexate (*GA: pcor = 0.053 and *GG: pcor = 0.053, respectively). However, the significant results were obtained for the dominant model as the best model of inheritance (GA+AA vs. GG, P = 0.032). In the similar sex-stratified analysis, a tendency was observed in boys with JIA for the rarer occurrence of the *PADI4* rs2240336*GG genotype in the case of the absence of the response to methotrexate (pcor = 0.091).

It was also noted that in the case of the absence of the response to methotrexate in patients with JIA, the *IL2-IL21 *rs6822844*TT and *LTA* rs909253*AG genotypes were nonsignificantly more common (*IL2-IL21 *rs6822844*TT: pcor = 0.054, *LTA* rs909253*AG: pcor = 0.098), and the statistically significant results were obtained for the *IL2-IL21* rs6822844 locus when testing the recessive model of inheritance (TT vs. GG+GT, P = 0.017).

### 3.2. The study of the predisposition to the insufficient response to methotrexate in JIA

Analysis of the *IL1B* rs16944 polymorphic locus showed a tendency towards a rarer occurrence of the *IL1B* rs16944*CC genotype in JIA patients with the insufficient response to methotrexate than in those with the sufficient (37.88% vs. 50.00%, respectively, pcor = 0.094) (Table 2). After stratification by sex, it turned out that boys with JIA, who did not achieve clinical remission on medication with the methotrexate therapy, had a significantly increased proportion of the *IL1B* rs16944*T allele (41.86% vs. 22.00%, P = 0.025, pcor = 0.024, OR = 2.553, 95% CI 1.195–5.410, in comparison with those who achieved, respectively). The log-additive model of inheritance reflected the results most successfully (2TT+CT vs. CC, P = 0.036). At the same time, there were no significant differences by the studied trait in girls with JIA (P > 0.1).

**Table 2:** The relationship of the polymorphic loci alleles and genotypes with the insufficient response to methotrexate in JIA patients.

Polymorphic loci			The whole group (f+m)				Female (f)				Male (m)				ni
Genes	rs	Alleles	Аlleles and genotypes frequencies, %		Model of inheritance		Аlleles and genotypes frequencies, %		Model of inheritance		Аlleles and genotypes frequencies, %		Model of inheritance		The total number of genotyped subjects, ni
			insufficient response sufficient response		var p	OR 95% CI	insufficient response sufficient response		var p	OR 95% CI	insufficient response sufficient response		var p	OR 95% CI	insufficient response sufficient response
		(1)/(2)	(2)	(11)/(12)/(22)			(2)	(11)/(12)/(22)			(2)	(11)/(12)/(22)			f+m : f : m
TNFA	1800629	G/A	7.2	85.6/14.4/0.0	-	-	7.3	85.4/14.6/0.0	-	-	7.0	86.0/14.0/0.0	-	-	132 : 89 : 43
			11.9	76.3/23.8/0.0			13.6	72.7/27.3/0.0			8.0	84.0/16.0/0.0			80 : 55 : 25
LTA	909253	A/G	27.7	50.8/43.2/6.1	NS	NS	25.3	55.1/39.3/5.6	NS	NS	32.6	41.9/51.2/7.0	NS	NS	132 : 89 : 43
			32.5	45.0/45.0/10.0			34.5	41.8/47.3/10.9			28.0	52.0/40.0/8.0			80 : 55 : 25
IL1B	16944	C/T	39.8	37.9/44.7/17.4	NS	NS	38.8	36.0/50.6/13.5	NS	NS	41.9	41.9/32.6/25.6	log-ad0.036	2.081.01-4.27	132 : 89 : 43
			31.9	50.0/36.3/13.8			36.4	43.6/40.0/16.4			22.0	64.0/28.0/8.0			80 : 55 : 25
IL2-21	6822844	G/T	9.5	83.3/14.4/2.3	NS	NS	7.9	85.4/13.5/1.1	NS	NS	12.8	79.1/16.3/4.7	NS	NS	132 : 89 : 43
			8.1	83.8/16.3/0.0			10.0	80.0/20.0/0.0			4.0	92.0/8.0/0.0			80 : 55 : 25
IL2RA	2104286	A/G	15.5	73.5/22.0/4.5	NS	NS	17.4	70.8/23.6/5.6	NS	NS	11.6	79.1/18.6/2.3	NS	NS	132 : 89 : 43
			18.1	67.5/28.8/3.8			18.2	67.3/29.1/3.6			18.0	68.0/28.0/4.0			80 : 55 : 25
IL6	1800795	G/C	40.2	33.3/53.0/13.6	NS	NS	41.0	29.2/59.6/11.2	NS	NS	38.4	41.9/39.5/18.6	NS	NS	132 : 89 : 43
			40.0	38.8/42.5/18.8			40.0	36.4/47.3/16.4			40.0	44.0/32.0/24.0			80 : 55 : 25
IL10	1800872	C/A	29.9	50.8/38.6/10.6	NS	NS	31.5	47.2/42.7/10.1	NS	NS	26.7	58.1/30.2/11.6	NS	NS	132 : 89 : 43
			28.1	52.5/38.8/8.8			24.5	56.4/38.2/5.5			36.0	44.0/40.0/16.0			80 : 55 : 25
MIF	755622	G/C	17.8	67.4/29.5/3.0	NS	NS	14.6	71.9/27.0/1.1	NS	NS	24.4	58.1/34.9/7.0	NS	NS	132 : 89 : 43
			21.9	63.8/28.8/7.5			21.8	61.8/32.7/5.5			22.0	68.0/20.0/12.0			80 : 55 : 25
CTLA4	3087243	G/A	33.0	44.7/44.7/10.6	NS	NS	30.3	49.4/40.4/10.1	NS	NS	38.4	34.9/53.5/11.6	dom0.0078	3.971.39-11.32	132 : 89 : 43
			26.3	56.3/35.0/8.8			29.1	50.9/40.0/9.1			20.0	68.0/24.0/8.0			80 : 55 : 25
NFKB1	28362491	I/D	47.3	33.3/38.6/28.0	over-d 0.0007	0.380.21-0.67	46.6	33.7/39.3/27.0	over-d0.0022	0.340.17-0.69	48.8	32.6/37.2/30.2	NS	NS	132 : 89 : 43
			43.8	25.0/62.5/12.5			43.6	23.6/65.5/10.9			44.0	28.0/56.0/16.0			80 : 55 : 25
PTPN22	2476601	G/A	12.5	77.3/20.5/2.3	NS	NS	12.9	77.5/19.1/3.4	NS	NS	11.6	76.7/23.3/0.0	NS	NS	132 : 89 : 43
			14.4	73.8/23.8/2.5			15.5	70.9/27.3/1.8			12.0	80.0/16.0/4.0			80 : 55 : 25
PADI4	2240336	G/A	43.2	29.5/54.5/15.9	NS	NS	43.3	30.3/52.8/16.9	NS	NS	43.0	27.9/58.1/14.0	NS	NS	132 : 89 : 43
			38.1	40.0/43.8/16.3			41.8	36.4/43.6/20.0			30.0	48.0/44.0/8.0			80 : 55 : 25

Note: (1) – the major allele, (2) – the minor allele; (11) and (22) – genotypes homozygous for the major and minor alleles, respectively; (12) – heterozygous genotype; f+m – female and male, f – female, m – male; Model of inheritance – the best model of inheritance (regression analysis): var – type of the model: dom – dominant: (12)+(22) vs (11); over-d – over-dominant: (12) vs (11)+(22); log-ad – log-additive: (22)+(22)+(12) vs (11); statistically significant results are highlighted in bold; NS – statistically nonsignificant.

For the alleles and genotypes of the *CTLA4* rs3087243 locus, associations with the insufficient response to methotrexate in the general JIA group and in girls with JIA have not been established (P > 0.1). At the same time, in boys with JIA with the insufficient response to methotrexate the *CTLA4* rs3087243*GA genotype and the *CTLA4* rs3087243*A allele were significantly more common, and the *CTLA4* rs3087243*GG genotype was significantly less common, than in those with the sufficient response (*GA: 53.49% vs. 24.00%, P = 0.023, pcor = 0.022, OR = 3.642, 95% CI 1.188–11.379; *A: 38.37% vs. 20.00%, P = 0.035, pcor = 0.036, OR = 2.491, 95% CI 1.128–5.553; *GG: 34.88% vs. 68.00%, P = 0.012, pcor = 0.013, OR = 0.252, 95% CI 0.094–0.733, respectively). The dominant model of inheritance described the results better than the others (GA+AA vs. GG, P = 0.0078).

Analysis of the *NFKB1* rs28362491 polymorphic locus showed that in JIA patients with the insufficient response to methotrexate the *NFKB1* rs28362491*DD genotype was significantly more common, and the *NFKB1* rs28362491*ID genotype was significantly less common, than in those with the sufficient response (*DD: 28.03% vs. 12.50%, P = 0.010, pcor = 0.011, OR = 2.726, 95% CI 1.306–5.568; *ID: 38.64% vs. 62.50%, P = 0.001, pcor = 0.0008, OR = 0.378, 95% CI 0.213–0.675, respectively). However, after stratification by sex, the same associations were observed only in girls with JIA (*DD: P = 0.022, pcor = 0.024, OR = 3.015, 95% CI 1.192–7.700; *ID: P = 0.003, pcor = 0.003, OR = 0.342, 95% CI 0.175–0.672, respectively). The best model of inheritance turned out to be the overdominant (ID vs. II+DD, P = 0.0007 and P = 0.0022 in the general JIA group and in girls with JIA, respectively).

For the separate variants of the *TNFA* rs1800629 and *LTA* rs909253 polymorphic loci, no associations with the insufficient response to methotrexate in JIA were found (P > 0.05). There was only a tendency towards an increase of the *TNFA* rs1800629*GG genotype proportion and a decrease of the *TNFA* rs1800629*GA genotype proportion in JIA patients who did not achieve clinical remission on medication with the methotrexate therapy (pcor = 0.096; the *AA genotype was absent in this sample). After stratification by sex, a similar trend for the *TNFA* rs1800629*GG and *GA genotypes was observed and even increased only in girls with JIA (pcor = 0.081).

A study of the haplotypes of the *TNFA* rs1800629 and *LTA* rs909253 loci showed a tendency towards a rarer occurrence of the A-G haplotype in JIA patients with the insufficient response to methotrexate than in those with the sufficient (7.20% vs. 11.87%, respectively, P = 0.082). In the similar sex-stratified analysis, these differences for the A-G haplotype increased and reached the statistical significance level, but only in girls with JIA (P = 0.046, OR = 0.41, 95% CI 0.18–0.98). 

For the alleles and genotypes of the *IL2-IL21* rs6822844, *IL2RA* rs2104286, *IL6* rs1800795,* IL10* rs1800872, *MIF* rs755622, *PTPN22* rs2476601, *PADI4* rs2240336 loci associations with the insufficient response to methotrexate were not observed (P > 0.1).

## 4. Discussion

As a result of the study, the predictors of the methotrexate efficacy in JIA were established. Associations with an altered risk of the nonresponse to methotrexate in JIA were observed for the alleles/genotypes of the loci* IL10* rs1800872 (in girls) and *NFKB1* rs28362491 (in girls); with an altered risk of the insufficient response to methotrexate in JIA - for the alleles/genotypes of the loci *IL1B *rs16944 (in boys), *CTLA4* rs3087243 (in boys), *NFKB1* rs28362491 (in girls), and the haplotype *TNFA* rs1800629*A - *LTA* rs909253*G (in girls) (Table 3).

**Table 3:** Genetic predictors of the methotrexate efficacy in JIA.

	Response to methotrexate					
	Absent			Insufficient		
	f+m	f	m	f+m	f	m
TNFA rs1800629 - LTA rs909253 (haplotypes)	-	-	-	-	A-G	-
IL1B rs16944	-	-	-	-	-	T, C
IL10 rs1800872	-	A, C, CC	-	-	-	-
CTLA4 rs3087243	-	-	-	-	-	GA, A, GG, G
NFKB1 rs28362491	DD, D, I	DD	-	DD, ID	DD, ID	-

Note: f+m – female and male; f – female; m – male; alleles and genotypes for which OR > 1 (P < 0.05) are highlighted in bold; underlined font on a gray background; alleles, genotypes, and haplotype for which OR < 1 (P < 0.05) are italicized.

According to the available data, the genetic markers considered in this work have not been previously studied for the associations with the methotrexate efficacy in JIA. 

It has been shown that the methotrexate treatment reduces the TNFα, IL1β, and IL6 production and increases the *IL10* expression in the synovial tissue of rheumatoid arthritis patients [12]. At the same time, Pawlik et al. (2006) established the interrelation of a number of the cytokine genes polymorphic loci variants with the level of phytohemagglutinin-stimulated secretion of the corresponding products by peripheral blood mononuclear cells of healthy volunteers from Poland [10]. In particular, there was a significant increase of the levels of IL6 in the *IL6* rs1800795*GG homozygotes (in comparison with the *IL6* rs1800795*CC homozygotes), of IL10 – in homozygotes for the GCC haplotype of the *IL10 *gene rs1800896, rs1800871, and rs1800872 loci (compared to the ATA/ATA, ACC/ACC, ACC/ATA genotypes), of TNFα – in the *TNFA* rs1800629*GA heterozygotes (in comparison with the *TNFA* rs1800629*GG homozygotes). The identified differences remained even after the treatment of the cell culture with increasing doses of methotrexate, but not dexamethasone [10]. Considering that IL10 is one of the main antiinflammatory mediators [11], these data are partially consistent with the results of the present work, where the association of the *IL10* rs1800872*A allele with an increased risk of the nonresponse to methotrexate in girls with JIA was shown. At the same time, TNFα has a powerful proinflammatory effect [11], but in the present work it was shown that the* TNFA* rs1800629*A allele in the* TNFA* rs1800629*A - *LTA* rs909253*G haplotype acts as a protective marker in relation to the insufficient response to methotrexate in girls with JIA. This fact may be due to the presence of the *LTA* rs909253*G allele, since Pociot et al. (1991) showed that the levels of the TNFα and IL1β production by lipopolysaccharide- and phytohemagglutinin-stimulated monocytes were significantly lower in healthy carriers of the *LTA* rs909253*AG genotype than in those with the *LTA* rs909253*AA genotype [29]. Given the sexual specificity of the established association, it can also be assumed that the observed contradictions are related to the peculiarities of the immune response regulation in males and females, leading to the differences in the secretion of cytokines and other mediators [30].

The data on the relationship of the *IL1B* rs16944 polymorphic loci variants with the *IL1B* expression are extremely contradictory. In particular, analyzing the* IL1B* gene haplotypes, Chen et al. (2006) showed the association of the *IL1B* rs16944*T allele with an increased transcriptional activity of the reporter gene when stimulated by lipopolysaccharide- and phorbol-12-myristate-13-acetate [31], and Wen et al. (2006) – with a decreased one when stimulated only by lipopolysaccharide [32]. In addition to the experimental features, these differences can also be associated with the samples characteristics, in particular with the sex ratio, since in the present work it has been shown that the *IL1B* rs16944*T allele increases the risk of the insufficient response to methotrexate only in boys with JIA. It should also be noted that the relationship of the *IL1B* rs16944*TT genotype with the nonachieving of the ACR Pedi 70 response to etanercept in JIA was previously established, which is important to consider when selecting the second-line therapy for these patients [33].

CTLA4 is a member of the immunoglobulin-related receptors family and is believed to play an important role in mediating the suppressive function of regulatory T cells [34]. Cribbs et al. (2015) showed that the methotrexate treatment of rheumatoid arthritis patients enhances the reduced *CTLA4* expression in regulatory T cells and restores their defective suppressor function [13]. Moreover, Kasela et al. (2017) found that the *CTLA4* rs3087243*A allele is associated with the higher *CTLA4* expression levels in the CD4+ and CD8+ T cells [35]. It can be assumed that the presence of the *CTLA4* rs3087243*A allele in boys with JIA indicates the predominance of other pathogenetic mechanisms that are not associated with the reduced *CTLA4* expression, which determines the absence of the important therapeutic target for the methotrexate exposure and, as a result, its insufficient efficacy in this group of patients.

An important role in the regulation of inflammation, cell survival and proliferation is played by NFκB, a family of transcription factors (RelA (p65), cRel, RelB, NFκB1 (p50 and its precursor p105), and NFκB2 (p52 and its precursor p100)), which functions as various hetero- or homodimers [12,36,37]. One of the key members of this family is p50 encoded by the *NFKB1* gene. It should be noted that p50 has opposite effects in the hetero-/homodimeric forms. Thus, p50/p50 homodimers have an antiinflammatory effect, inhibiting the transcription of proinflammatory cytokine genes, including *TNFA*, and enhancing the *IL10 *gene transcription. At the same time, p50/p65 heterodimers exhibit proinflammatory properties and stimulate the transcription of the corresponding cytokine genes, including *TNFA* [36–38]. Methotrexate has been shown to inhibit the p65 phosphorylation and thereby to downregulate the expression of *TNFA* [39,40]. It is assumed that the *NFKB1* rs28362491*D allele may cause a decrease in the *NFKB1* expression and, accordingly, the p50 production; the decrease in the p50/p50 homodimer levels most likely exceeds the decrease in the p50/p65 heterodimer levels, which leads to the increased proinflammatory cytokine production in these patients [37,38,41]. In addition, the decrease in the p50/p65 heterodimer formation may lead to the partial loss of an important therapeutic target for the methotrexate exposure, which overall mediates its subsequent inefficiency. At the same time, an increased proinflammatory cytokine production in the *NFKB1* rs28362491*D allele carriers may suggests a good effect of an anticytokine therapy. This hypothesis is confirmed by the data of Nazarova et al. (2018), according to which the *NFKB1* rs28362491*D allele reduces the risk of nonachieving the ACR Pedi 70 response to etanercept in JIA patients [33]. At the same time, in the present work it was shown that the *NFKB1* rs28362491*DD genotype marks an increased susceptibility to the nonresponse to methotrexate in JIA (in girls). These data are of particular interest in terms of predicting the efficacy of JIA therapy: they allow to identify the risk group for the nonresponse to methotrexate and at the first signs of an unfavorable prognosis realization or initially to recommend the strengthening of therapy by prescribing etanercept for this group.

The potential limitations of this research are the relatively small number of the studied polymorphic genes variants, as well as the possible ethnic specificity of the identified associations. To confirm the obtained data, replicative studies on different ethnicity samples are required, as well as additional immunological studies to assess the true causes underlying the observed associations. In addition, the prediction of the JIA therapy efficacy using the genetic testing is quite expensive, so the implementation of this method in low economic communities may be rather difficult.

This study was aimed to identify the potential predictors of the JIA patients’ response to methotrexate among some of the immune response mediator genes’ polymorphic loci (*TNFA* rs1800629, *LTA* rs909253, *IL1B *rs16944, *IL2-IL21* rs6822844, *IL2RA* rs2104286, *IL6* rs1800795, *IL10* rs1800872, *MIF* rs755622, *CTLA4* rs3087243, *NFKB1* rs28362491, *PTPN22* rs2476601, *PADI4* rs2240336) variants. As a result of the study, the relationship of the alleles/genotypes of the *IL1B *rs16944, *IL10* rs1800872, *CTLA4* rs3087243, *NFKB1* rs28362491 polymorphic loci and the *TNFA* rs1800629*A - *LTA* rs909253*G haplotype with the methotrexate efficacy in JIA has been established (taking into account the differences by sex). Associations for the *IL1B* rs16944 and *NFKB1* rs28362491 polymorphic loci variants seem to be the most promising, since they allow to evaluate the efficacy prognosis simultaneously for two the most widely used disease-modifying antirheumatic drugs in the JIA treatment: methotrexate and etanercept. If confirmed in replicative studies, the established markers might be used for the early prediction of the JIA therapy efficacy and the timely appointment of an adequate medication, which is crucial for preventing the disease complications.

## Acknowledgments

The work was supported by:

1) Government project: “Study of molecular genetic mechanisms of formation of multifactorial pathology”, No. 115060810015 (08 June 2015).

2) Grant of the Republic of Bashkortostan to young scientists and youth scientific teams, contract No. 6 (25 March 2016).

3) The program “Participant of the Youth Scientific and Innovation Contest” (“UMNIK”), contracts No. 10/16859 (28 May 2012) and No. 10/20810 (01 July 2013).

## Conflict of interest

The authors declare that they have no conflict of interest.
